# The Report of the Committee on the Inebriates Acts—I

**Published:** 1909-01-16

**Authors:** 


### SPECIAL ARTICLE.

### THE REPORT OF THE COMMITTEE ON THE INEBRIATES ACTS—I.

The Departmental Committee on the Inebriates Acts has presented its Report with unusual celerity. Appointed at the end of April last, it produces in December a seasoned Report of 39 pages concluding with 40 recommendations and accompanied by 235 folio pages of evidence. Such an achievement constitutes, we believe, a "record" in the history of Departmental Committees. Nor is there in the Report any evidence of undue haste or precipitancy in considering the evidence and arriving at conclusions. The Recommendations are of great and far-reaching importance, and some of them are quite novel; but they rest on a considerable body of evidence, are supported by cogent arguments, and must command attentive consideration.

The terms of reference were as follows:—"To inquire into the Law relating to Inebriates, and to their detention in Reformatories and Retreats, and to report what amendments in the law and its administration are desirable." This original reference was, on August 7, extended "To investigate the value of existing methods for the treatment of inebriety by the use of drugs."

The attention of the medical profession will naturally be attracted in the first place to the second reference, and will look with some curiosity to the manner in which it has been treated by the Committee. It is obvious that a body consisting of four doctors, not one of whom is a pharmacologist, and four lawyers, with a county magnate for chairman, is not an ideal body for the determination of the value of modes of medical treatment by drugs. Any pronouncement on the question, by such a body, would not command the confidence of the medical profession; and the value of their conclusion, whatever it might be, discounted beforehand by the constitution of the inquiring body, would be still further discredited if it had been arrived at in the short period of eight months. Whether the Committee itself was influenced by these considerations we do not know, but if it had been sensible of them, it could scarcely have avoided the difficulty more completely or more adroitly. Into the efficacy or the comparative merits of these modes of treatment it has not inquired at all. It has confined its investigations into the effect that such modes of treatment, supposing they possessed all the virtue and all the efficacy claimed by their advocates, would have upon the existing system of treating inebriates by terms of detention enforced by law; and it decides that they would have no effect at all. The reasons given for this conclusion are overwhelming. The Committee shows, first, that it would be impracticable to set forth in an Act of Parliament the various modifications of any specific treatment that must be required by the varying needs of individual cases; second, that the fate of the Vaccination Acts shows that no mode of medical treatment can be enforced by Act of Parliament without producing more friction, discontent and agitation than it is worth; third, that it would be unjustifiable to enforce on the voluntary inmates of Retreats a specific mode of treatment to which they might object; fourth, that, if administered while the inebriate was under detention, the authorities of Reformatories would be tied down to one mode of treatment instead of being free, as they now are, to adopt any method, proprietary or non-proprietary, they choose; fifth, that if administered to offenders at large, the inebriate would have to give "recognisances to come up for treatment so many times a day for a specified period or until the proper authority considered him cured." The Committee remark naïvely that "the administrative difficulties in the way of this procedure would be considerable, and its novelty might excite prejudice against it." Sixth, the Committee finds that the worst cases of all those, namely, who do not desire to be cured, and therefore do not voluntarily submit themselves to treatment, are not attempted by the drug curers, and therefore there is no evidence whether they can be so cured or not.

The Committee, therefore, makes no recommendation for the compulsory application of any drug cure to inebriates in retreats and reformatories; and as to the efficacy or comparative merits of the various drug cures it has made no investigation, and deprecates any investigation being made. The Committee says, very justly, that such an investigation would involve a great expenditure of time and money. It certainly would. The Committee would have to watch for years the effect of administering an unknown number of "cures" to an indefinite number of inebriates; would have to satisfy itself whether or no each inebriate abandoned his habit; and whether, in what time, and to what degree, he returned to it. The investigation of the Tuberculosis Commission would be a bagatelle in comparison. Such an expenditure of time and money, on such an object, the Committee think would not be justified unless there were a strong public demand for it. We are of opinion that it would not be justifiable under any circumstances; for it would be impossible for any Committee to arrive at results sufficiently conclusive to be commensurate with the time and money expended. A further reason given by the Committee for not pursuing these investigations is really superfluous in view of all that it has said before, but yet is by no means to be overlooked. Such an inquiry would be cited as a precedent by the proprietors of other "cures" for other diseases, as a reason for investigating the efficacy of their nostrums, and we should have the edifying spectacle of Government inquiries into the Bartitsu light cure, electric belts, anti-rheumatic bracelets, Cockle's pills, and Holloway's ointment, and so forth. It is manifest that the inquiry into these "cures" for inebriety ought never to have been granted; but, having been granted, it is unexpectedly fortunate that it has fallen into the hands of a Committee that recognised its absurdity and dealt with it as it deserved.

